# Curcumin in solid organ transplantation: current evidence, mechanisms, and translational challenges

**DOI:** 10.3389/fphar.2026.1911685

**Published:** 2026-10-08

**Authors:** Lubiao Liang, Wei Xiong, Chunyang Guo, Yongxiang Song, Yajin Zhao

**Affiliations:** 1 Department of Thoracic Surgery, Affiliated Hospital of Zunyi Medical University, Zunyi Medical University, Zunyi, Guizhou, China; 2 Key Laboratory of Oral Disease Research of the Education Department of Guizhou Province, Stomatology Hospital of Zunyi Medical University, Zunyi, Guizhou, China

**Keywords:** curcumin, organ transplantation, ischemia-reperfusion injury, natural products, bioavailability

## Abstract

Solid organ transplantation faces complex pathophysiological challenges, including ischemia-reperfusion injury (IRI), alloimmune rejection, and chronic graft dysfunction. These interconnected processes involve multiple signaling pathways, yet these complex interconnected pathological processes remain a significant clinical challenge. In contrast, natural plant-derived compounds often possess pleiotropic pharmacological activities and may offer a multi-targeted approach to modulate the overall pathological milieu. Curcumin, a representative bioactive natural polyphenol from *Curcuma longa*, exhibits potent antioxidant, anti-inflammatory, and immunomodulatory properties and has shown therapeutic benefits across a wide range of diseases. In the context of transplantation, preliminary studies have demonstrated encouraging effects in improving graft preservation, attenuating injury, and modulating immune response. However, direct investigation of curcumin in solid organ transplantation remains limited, with fragmented evidence and a weak foundational basis. This review aims to systematically synthesize the current evidence for curcumin’s application in kidney, liver, heart, and lung transplantation, to assess the translational hurdles, and to propose a strategic roadmap for future research. We also propose a mechanistic framework for curcumin’s potential actions based on established studies in related fields, including IRI, inflammatory diseases, and immunology, intended to generate testable hypotheses for future transplant-focused research. By identifying key knowledge gaps and highlighting promising mechanistic pathways, we hope to stimulate further investigation and accelerate the clinical translation of this pleiotropic natural compound into transplant medicine.

## 1 Introduction

Organ transplantation is a standard treatment for end-stage organ failure, improving patient survival and quality of life (WHO, Transplantation Society (TTS), and Organización Nacional de Trasplantes (ONT), 2011). However, its success is limited by multiple factors, including organ shortage, suboptimal donor organ quality, ischemia-reperfusion injury (IRI), and complications from lifelong immunosuppressive therapy (Zhao et al., 2023). To address these challenges, there is a continuous search for adjunctive agents that can enhance graft protection, reduce required immunosuppressant doses, and minimize adverse effects. However, effective management of these complex and interconnected pathological processes remains an unmet clinical need.

In this context, natural plant-derived compounds, which often possess pleiotropic pharmacological activities and favorable safety profiles, offer a potential multi-targeted alterative. Curcumin, a representative bioactive polyphenolic compound extracted from the rhizome of turmeric (*Curcuma longa*), has garnered considerable attention. Curcumin possesses multifaceted pharmacological properties, such as antioxidant, anti-inflammatory, and immunomodulatory activities, alongside with a favorable safety profile and low cost (Wu, 2003; Sharma et al., 2005; Jin, 2018; Jin et al., 2018). These properties have driven extensive research across diverse disorders, such as cancer (Mohankumar et al., 2021; Giordano and Tommonaro, 2019), sepsis (Zhong et al., 2016), SARS-CoV-2 infection (Marín-Palma et al., 2021), hepatosteatosis (Chen et al., 2017; Maithilikarpagaselvi et al., 2016), atherosclerosis (Ghosh et al., 2014), diabetes, and obesity (Jin et al., 2018; Weisberg et al., 2008; Shao et al., 2012), with clinical studies also reporting effects in major depression (Lopresti and Drummond, 2017; Yu et al., 2015), non-alcoholic fatty liver disease (Rahmani et al., 2016), osteoarthritis (Panahi et al., 2016), type 2 diabetes (Chuengsamarn et al., 2014), solid tumors (Panahi et al., 2014), and COVID-19 (Pawar et al., 2021).

Of relevance to transplantation, curcumin has been shown in animal models to attenuate IRI across multiple organs by modulating oxidative stress, inflammation, and cell death (Kar et al., 2020; Wang et al., 2017; Li et al., 2015) with clinical data suggesting potential protective effects (Wongcharoen et al., 2012). Several reviews have summarized curcumin’s impact on IRI (Mohamadian et al., 2022; Teng et al., 2022; Mauerhofer et al., 2021; Bavarsad et al., 2019), a key driver of early graft dysfunction. However, solid organ transplantation represents a uniquely complex clinical scenario that extends beyond IRI to encompass alloimmune responses, organ preservation injury, and interactions with chronic immunosuppression.

Therefore, this review aims to address this gap by systematically evaluating the available evidence on curcumin’s application in kidney, liver, heart, and lung transplantation. We examine its role in donor organ preservation, IRI mitigation, immunomodulation, and potential interactions with conventional immunosuppressants. Key mechanisms are synthesized, and the principal challenges that have constrained both research progress and clinical translational are discussed to chart a path forward for this promising but underexplored area.

## 2 Biology of curcumin

Curcumin (diferuloylmethane) is an orange-yellow crystalline polyphenol and the principal bioactive constituent of the dietary spice turmeric (*Curcuma longa* L.). This natural pigment, chemically defined as 1,7-bis(4-hydroxy-3-methoxyphenyl)-1,6-heptadiene-3,5-dione (C_21_H_20_O_6_), is a low-molecular-weight, lipophilic diarylheptanoid. Its characteristic structure—featuring two o-methoxyphenol rings connected by a seven-carbon β-diketone chain—enables keto-enol tautomerism and confers potent free-radical scavenging activity (El-Saadony et al., 2025; Urosevic et al., 2022), which underpins its broad pharmacological profile. Curcumin is practically insoluble in aqueous media at neutral or acidic pH but is readily soluble in organic solvents such as dimethyl sulfoxide, ethanol, methanol and acetone (Priyadarsini, 2014). It is chemically unstable at physiological to alkaline pH (≥6.5) and under visible/UV light, undergoing rapid autoxidative degradation to products like vanillin, ferulic acid and cyclization/dimerization derivatives, which contributes to its poor stability and very low oral bioavailability (Kaur et al., 2024). After ingestion, curcumin is extensively metabolized in the intestine and liver through reductive and phase II conjugation reactions (glucuronidation and sulfation), resulting in only trace plasma levels of the native compound (Cerullo et al., 2025; Tian et al., 2025). Despite these pharmacokinetic and physicochemical limitations, the aromatic-phenolic and β-diketone moieties of curcumin mediate a broad spectrum of bioactivities, including antioxidant, anti-inflammatory, immunomodulatory, metabolic-regulatory and anti-fibrotic activities across preclinical models (Liu et al., 2025; Memarzia et al., 2021) (Figure 1). These pleiotropic, multi-target actions have driven its investigation in inflammatory, metabolic, and degenerative diseases and are relevant to organ transplantation, where they intersect with key pathogenic axes of IRI, alloimmune response, and chronic graft fibrosis.

**FIGURE 1:**
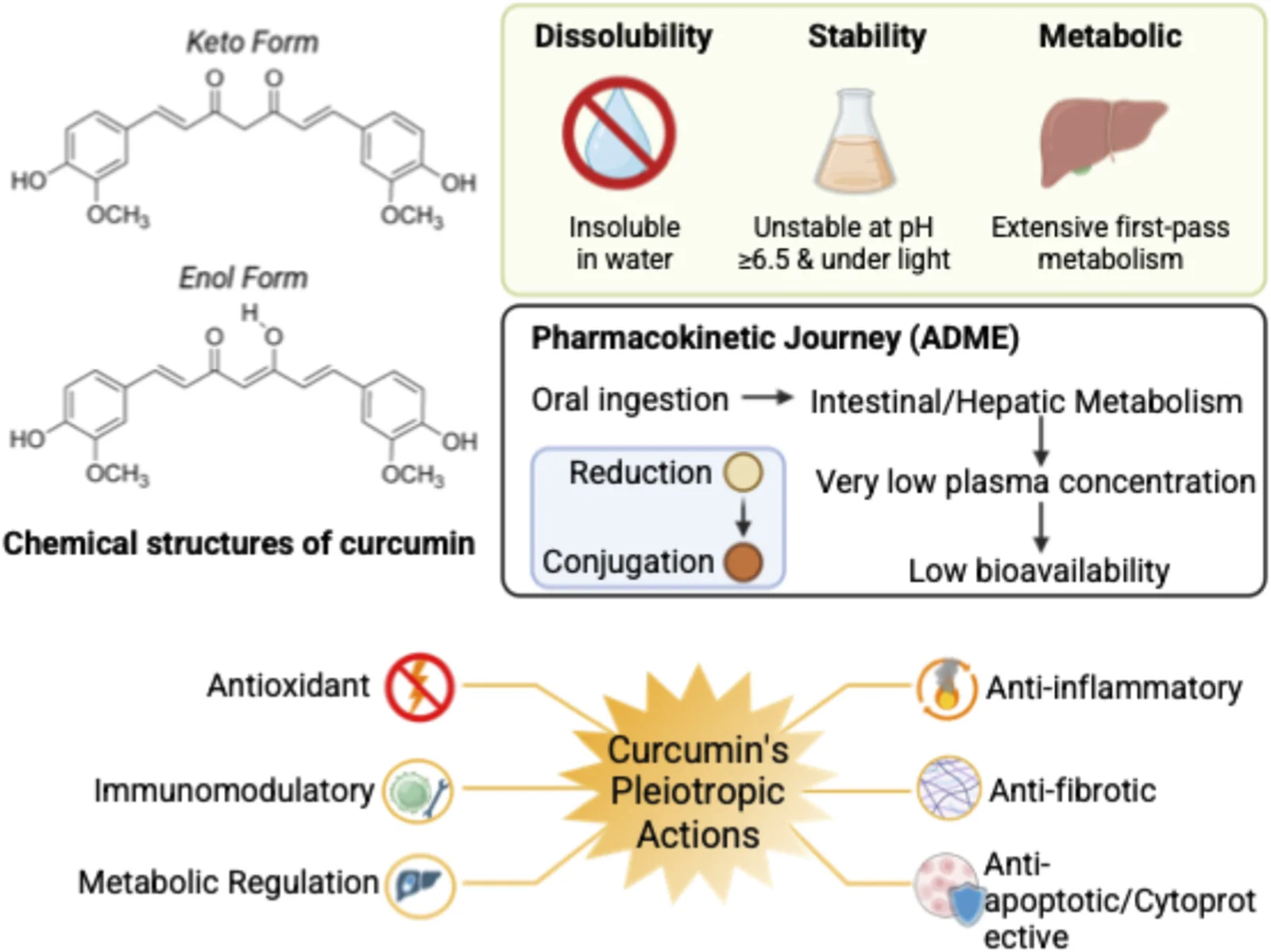
Chemical and biological profile of curcumin. Created with BioRender.com.

## 3 Curcumin in solid organ transplantation

The potential of curcumin to enhance donor organ preservation, mitigate IRI-induced graft dysfunction, and modulate allograft rejection has been explored across various solid organ transplantation settings (Table 1).

**TABLE 1:** Summary of preclinical and clinical evidence for curcumin in solid organ transplantation.

Organ	Models & findings	Observations	Limitations & unresolved questions
Kidney	Clinical: A Phase I/II trial reported that a curcumin-quercetin supplement was well-tolerated, with observed improvements in early graft function in a subset of patients with DGF (Shoskes et al., 2003; Shoskes et al., 2005) (Shoskes DA and Wee, 2007) Preclinical: Porcine autologous DCD transplant model demonstrates that a cyclodextrin-curcumin complex (CDC) accelerates functional recovery and attenuates fibrosis (Thuillier et al., 2014). Rat model shows synergy with bone marrow-derived mesenchymal stem cells (BM-MSCs) (Zhang et al., 2015). *Ex vivo* human PBMC studies show inhibition of IFN-γ production (Bharti et al., 2010)	1. Represents the only organ with reported human trial data in transplantation 2. Large-animal data suggest potential effects on both acute injury and chronic pathology 3. Provides proof-of-concept for immunomodulatory activity in human immune cells	1. Human data derive from a combination product; curcumin’s individual role is unclear 2. The porcine model uses autotransplantation, leaving allogeneic efficacy untested 3. Optimal dose, formulation, and pharmacokinetics in transplant patients are unknown
Liver	Preclinical: Cellular/*ex vivo* models show endothelial protection (Fuller et al., 2003), HO-1 induction (McNally et al., 2006). Addition to preservation solutions improves perfusion (Chen et al., 2006) and mitigates oxidative stress in DCD models (Saidi et al., 2018). An orthotopic mouse transplant model elucidates the Kupffer cell PPARγ/NF-κB mechanism (Liu et al., 2018). Multi-drug preconditioning is effective (Moussavian et al., 2011). Negative Data: CDC failed to show overall benefit in a rigorous porcine DCD liver transplant model (Meurisse et al., 2024)	1. Mechanistically the most explored organ, with studies spanning multiple injury phases 2. Highlights the potential influence of organ-specific metabolism (liver vs. kidney) on outcomes 3. Underscores the discrepancy between promising rodent data and neutral results in a clinically relevant large-animal model	1. No clinical studies have been conducted 2. Translation of effective pretreatment regimens from rodents to clinical donor management is challenging 3. The reasons for the divergent outcomes between kidney and liver pig models require clarification
Heart	Preclinical: Rat heterotopic allograft models show monotherapy and synergy with sub-therapeutic cyclosporine prolong graft survival (Chueh et al., 2003). Human cardiomyoblast model shows CDC protects against cold storage injury via HO-1 induction (Hadil Abuarqoub et al., 2007)	1. Provides direct evidence of immunomodulation and drug synergy 2. Preliminary exploration of use in cardiac preservation	1. Evidence is very limited and model-dependent. 2. Lacks organ-level preservation studies and more complex transplant models. 3. No clinical data
Lung	Preclinical: A single rat orthotopic transplant study demonstrates pretreatment attenuates IRI, inhibits NF-κB and MMP-9 (Sun et al., 2008)	1. Serves as a proof-of-concept for efficacy against lung IRI in a transplant setting	1. Represents a major evidence gap, with only one study published over 15 years ago. 2. No research on preservation solutions or chronic rejection 3. No clinical data

Abbreviations: DGF, delayed graft function; DCD, donation after circulatory death; CDC, cyclodextrin-complexed curcumin; BM-MSC, bone marrow-derived mesenchymal stem cell; PBMC, peripheral blood mononuclear cell; IRI, ischemia-reperfusion injury.

### 3.1 Curcumin in kidney transplantation

Renal transplantation represents the most advanced frontier for curcumin research, uniquely bridging preclinical investigation with preliminary clinical evaluation. The evidence spans from cellular immunology and large-animal models to early-phase human trials, though significant limitations remain.

Following early investigation into the protective effects of curcumin and related flavonoids in renal IRI (Shoskes, 1998) and skin allograft rejection (Shoskes and Shahed, 2000), Shoskes et al. conducted a Phase I study to evaluate an oral supplement containing curcumin (400 mg) and quercetin (100 mg) in kidney transplant recipients. The therapy proved safe and well-tolerated. In patients with delayed graft function, supplementation was associated with improved early urine output and serum creatinine recovery, correlating with reduced lipid peroxidation (Shoskes et al., 2003; Shoskes et al., 2005). Long-term follow-up suggested potential benefits in graft survival and acute rejection rates (Shoskes DA and Wee, 2007). However, these clinical findings pertain specifically to the curcumin-quercetin combination. The individual contribution of curcumin, its optimal dose, and pharmacokinetics in transplant patients remain undefined.

At the cellular level, *ex vivo* treatment with curcumin suppressed IFN-γ production in activated peripheral blood lymphocytes from transplant patients, an effect linked to inhibition of the NF-κB pathway (Bharti et al., 2010). This provides a direct human immune-cell-based rationale for curcumin’s immunomodulatory potential.

In a porcine autologous donation after circulatory death (DCD) kidney transplant model, the addition of a cyclodextrin-complexed curcumin formulation (CDC) to the preservation solution showed compelling results. Treated grafts demonstrated accelerated functional recovery, reduced histological injury, and decreased infiltration of both innate and adaptive immune cells. Furthermore, curcumin treatment attenuated markers of interstitial fibrosis, tubular atrophy, and chronic inflammation at 3 months (Thuillier et al., 2014). While these results point to a protective effect of curcumin in kidney transplantation, the same CDC formulation and a similar dosing strategy failed to show significant overall benefit in a porcine DCD liver transplantation model (Meurisse et al., 2024). This discrepancy may underscore the potential for organ-specific differences in curcumin’s efficacy or metabolism.

Further expanding its potential, curcumin has been explored in combination with other immunomodulatory agents. In a rat kidney transplant model, co-administration of oral curcumin with bone marrow-derived mesenchymal stem cells (BM-MSCs) synergistically enhance the suppression of alloimmune responses. This combination led to significant reductions in serum pro-inflammatory cytokines (IL-2, IL-6) and graft expression of the pro-fibrotic factor TGF-β1 compared to BM-MSC therapy alone (Zhang et al., 2015). This suggests curcumin may serve as a beneficial adjunct, potentially amplifying the efficacy of cellular or other immunomodulatory therapies in transplantation.

Despite representing the most clinically advanced organ for curcumin research, the evidence in kidney transplantation remains preliminary. The only human data derive from a combination product, precluding conclusions about curcumin alone. The promising large-animal findings, while mechanistically informative, come from an autologous model that does not address alloimmunity. Thus, even in leading organ, the evidence base is fragmentary and require further validation.

### 3.2 Curcumin in liver transplantation

Research on curcumin in liver transplantation provides relatively the most extensive exploration, though evidence remains preclinical.

Initial studies focused on endothelial protection. Using proinflammatory vascular effluent from human donor livers, Fuller et al. demonstrated that curcumin significantly attenuated the cold hypoxia-induced upregulation of adhesion molecules ICAM-1 and E-selectin on endothelial cells (Fuller et al., 2003). Subsequently, the protective effect of curcumin pretreatment on 4 °C cold preservation-induced hepatic injury was examined. Curcumin administrated before cold storage was found to induce heme oxygenase-1 (HO-1), a key stress-response protein, during simulated cold preservation and warm reperfusion, with effects sustained over 48 h of storage (McNally et al., 2006). While this provided a mechanistic basis for extending preservation times, the required 6-h pretreatment poses a clinical challenge.

To address this, strategies evolved to incorporate curcumin directly into preservation solutions. Chen et al. added curcumin to various preservation solutions (Euro-Collins, phosphate buffer saline, and University of Wisconsin solution) and found improved portal flow, bile production, and hepatocellular integrity in isolated rat livers, extending the viable preservation window beyond 36 h (Chen et al., 2006). Saidi et al. studied curcumin in DCD liver preservation using CDC to enhance stability, which mitigated oxidative stress associated with combined warm ischemia and cold storage (Saidi et al., 2018).

The most significant mechanistic advance came from an orthotopic mouse liver transplant model. Donor pretreatment with curcumin reduced post-transplant inflammation and apoptosis and improved survival. These benefits were mechanistically linked to modulation of Kupffer cell function, particularly through PPARγ-related pathways (Liu et al., 2018). This study connected a specific immunomodulatory action within liver-resident macrophages to improved transplant outcomes. Furthermore, a multi-drug preconditioning regimen containing curcumin showed synergistic benefits in reducing inflammation and cell death in an isolated liver model, highlighting its potential role within combination therapies (Moussavian et al., 2011).

While the preclinical data are promising, a recent study underscores the complexity of translating curcumin into clinical practice. In a porcine model of DCD liver transplantation, Meurisse et al. (2024) evaluated the addition of the same water-soluble CDC (60 μM) to the preservation and flush solution (Meurisse et al., 2024). Contrary to expectations from rodent studies and positive results in a porcine DCD kidney model (Thuillier et al., 2014), the intervention did not significantly reduce serum AST levels, improve most markers of liver function, or enhance recipient survival. A potential signal—reduced incidence of primary nonfunction and lower serum lactate at 3 h post-reperfusion—did not translate into overall benefit. Several factors may explain the discrepancy. First, the liver is the primary site of curcumin’s rapid metabolism, likely reducing local availability during reperfusion (Sharma et al., 2007). Second, the single-dose preservation strategy may be pharmacologically insufficient compared to the multi-dose pre-conditioning used in rodent studies. Third, the injury-therapy window in large animal models requires precise calibration. This discrepancy emphasizes that efficacy in transplantation is organ-dependent and cannot be extrapolated from small animal data alone, calling for organ-specific translational study designs. Taken together, this negative large-animal study serves as a critical counterbalance to the predominantly positive preclinical literature and underscores the importance of reporting and interpreting neutral findings to guide future research.

### 3.3 Curcumin in heart transplantation

Evidence for curcumin in heart transplantation, though limited, provides proof of its immunomodulatory potential and hints at its utility in organ preservation. In rat heterotopic allogeneic cardiac transplant models (BN-to-WKY and BUF-to-WF), curcumin monotherapy significantly prolonged mean graft survival time. When synergy with sub-therapeutic doses of cyclosporine (CsA), it led to extended or even long-term graft acceptance. This benefit was associated with reduced expression of IL-2, IFN- γ and granzyme B (Chueh et al., 2003). These findings suggest that a potential of curcumin as an adjunctive immunomodulator.

Subsequently, Hadil et al (Hadil Abuarqoub et al., 2007). investigated curcumin’s role in mitigating cold storage injury using a human cardiac myoblasts model. They found that curcumin added during cold preservation in Celsior solution markedly attenuated cell death and metabolic dysfunction during subsequent rewarming. This protective effect was partially dependent on HO-1 induction, as inhibiting HO-1 activity attenuated the benefit. Curcumin also protected cells against damage during cold storage alone (without rewarming), suggesting potential utility as an additive to cardiac preservation solutions (Hadil Abuarqoub et al., 2007).

The evidence in heart transplantation is sparse and conceptual. The immunomodulatory synergy with cyclosporine comes from a single study using a heterotopic model that does not fully recapitulate clinical transplantation. The preservation-related data are limited to cell culture. Overall, the field lacks organ-level studies, and no clinical data exist.

### 3.4 Curcumin in lung transplantation

Research on curcumin in lung transplantation is limited, with one pivotal study providing comprehensive evidence. In a rat orthotopic lung transplant model, Sun et al. (2008) demonstrated that pretreatment with curcumin (200 mg/kg, intraperitoneal) in both donors and recipients conferred significant protection against IRI (Sun et al., 2008). Curcumin pretreatment attenuated the decline in PaO_2_/FIO_2_ ratio, reduced lung edema, and decreased alveolar-capillary leakage. These improvements were corroborated by histology, showing reduced inflammatory cell infiltration and hemorrhage. Mechanistically, the protection was linked to the suppression of the NF-κB pathway. Curcumin inhibited NF-κB activation in lung grafts, leading to significant downregulation of pro-inflammatory mediators (TNF-α and IL-6) at both mRNA and protein levels. Furthermore, curcumin treatment inhibited the activity of matrix metalloproteinase-9 (MMP-9), an enzyme implicated in barrier disruption and tissue remodeling. This study remains the most complete mechanistic investigation of curcumin in lung transplantation, establishing a possible rationale for its use. However, no research over a decade has expanded on these findings. There is no published research investigating curcumin as an additive to clinical lung preservation solutions. Moreover, its impact on lung-specific alloimmune pathologies remains completely unexplored. Therefore, while existing data suggest potential, lung transplantation represents a critical evidence gap and a high-priority area for future study.

## 4 Possible mechanisms of curcumin in organ transplantation

While curcumin has demonstrated preliminary efficacy across multiple solid organ transplantation models, understanding of its mechanisms within this specific context remains limited. Much of the mechanistic understanding derives from broader studies on IRI and inflammatory disease. Accordingly, this section synthesizes the established evidence to propose an integrated mechanistic framework for curcumin’s protective and immunomodulatory actions in solid organ transplantation. By doing so, we seek to highlight potential molecular targets and hypotheses to guide future investigation in this field.

### 4.1 Protective mechanisms against IRI

IRI is an inevitable process in organ transplantation, contributing to primary graft dysfunction and increased rejection risk. Curcumin is proposed to attenuate IRI through the following multi-targeted mechanisms, thereby providing initial protection to the graft (Figure 2).

**FIGURE 2:**
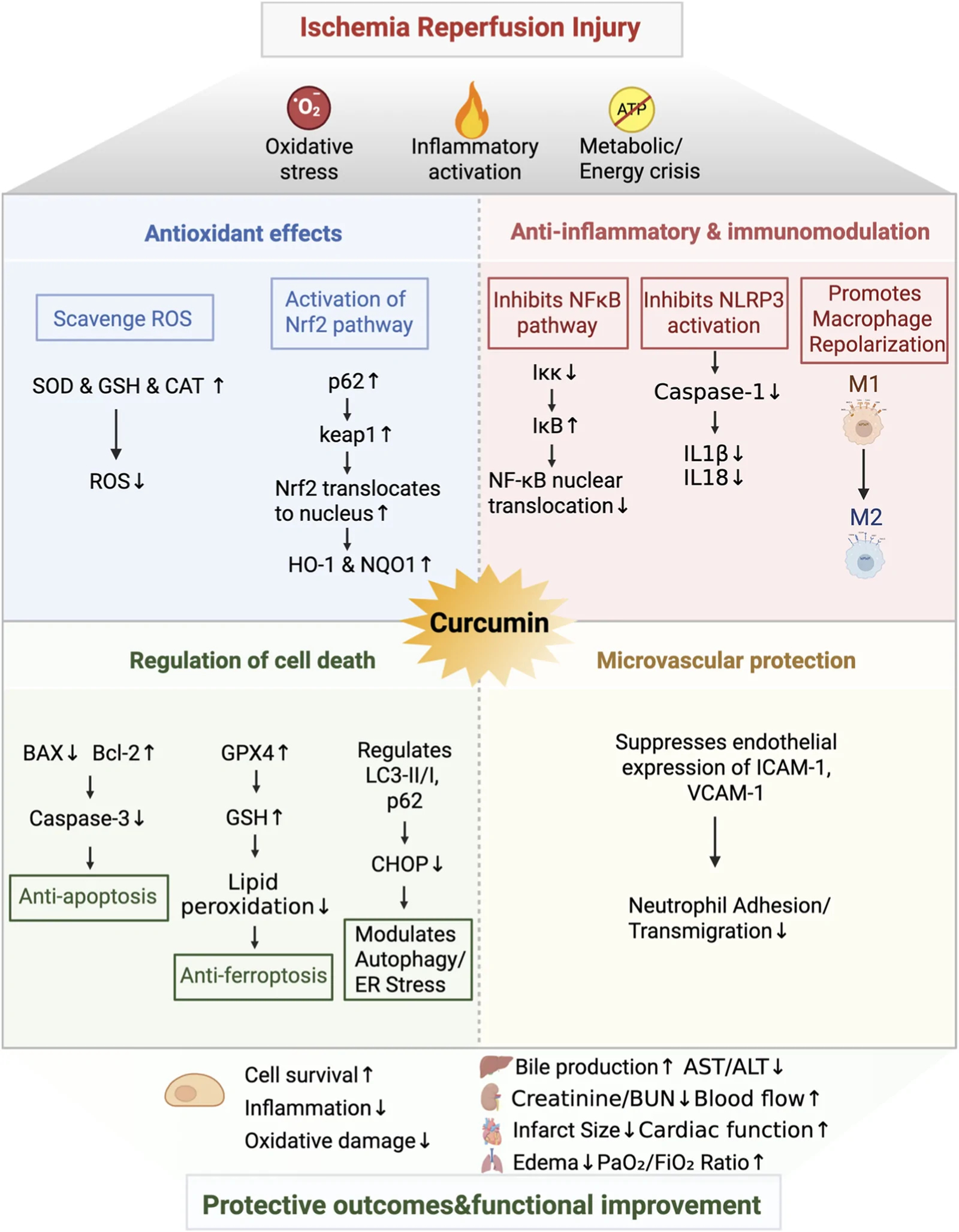
Potential multi-target protective mechanisms of curcumin against ischemia-reperfusion injury in transplantation. The upper panel summarizes the three major upstream pathological events of IRI—oxidative stress, inflammation activation, and metabolic/energy crisis. The middle panel illustrates curcumin’s proposed mechanisms of action: antioxidant effects, anti-inflammatory effects, regulation of cell death, and microvascular protection. The lower panel indicates the downstream functional outcomes of these protective effects, including improved organ function in the liver, kidney, heart, and lung. By concurrently targeting these pathways, curcumin promotes cellular protection and organ functional recovery. Created with BioRender.com.

#### 4.1.1 Antioxidant effects and activation of the Nrf2 pathway

Curcumin has been demonstrated to directly scavenge ROS and inhibit lipid peroxidation. This is supported by findings of decreased malondialdehyde (MDA) levels and elevated activity of endogenous antioxidant enzymes such as superoxide dismutase (SOD), glutathione (GSH), glutathione peroxidase (GPx), and catalase (CAT) in various IRI models (Liu et al., 2017; He et al., 2020; Cui et al., 2021; Zha et al., 2021; Yuan et al., 2024; Wang N. et al., 2025). Beyond direct scavenging, curcumin is reported to potently activate the nuclear factor erythroid 2–related factor 2 (Nrf2) antioxidant response pathway. By modulating the p62/Keap1/Nrf2 axis, it enhances the transcription of downstream cytoprotective enzymes, including HO-1 and NAD(P)H quinone oxidoreductase 1 (NQO1) (Balogun et al., 2003; Wu et al., 2013; Li et al., 2016; Lin et al., 2019; Ibrahim et al., 2020; Li X. et al., 2023). This coordinated antioxidant defense is crucial for protecting vulnerable endothelial and parenchymal cells from oxidative insult.

#### 4.1.2 Anti-inflammatory actions and immune cell modulation

Studies have shown that curcumin exerts anti-inflammatory effects primarily by inhibiting signaling hubs including NF-κB (Li et al., 2016; Li et al., 2021) and c-Jun N-terminal kinase (JNK) in IRI (Fiorillo et al., 2008; Ran et al., 2021). By activating JAK2/STAT3 signaling pathway, curcumin inhibits NF-κB mediated inflammation in IRI (Zhang J. et al., 2018), and curcumin protects IRI-induced apoptosis and inflammation by inhibiting activation of the Wnt/JNK1 signaling pathway (Zhou et al., 2020). These actions lead to significant downregulation of pro-inflammatory cytokines and chemokines, such as TNF-α, IL-1β, IL-6, and IL-8, and reduced activity of myeloperoxidase (MPO), attenuating neutrophil and macrophage infiltration into the injured tissue (Mohamadian et al., 2022; Cui et al., 2021; Ibrahim et al., 2020). Furthermore, curcumin modulates the crosstalk between the Nrf2 and NF-κB pathways and inhibits the activation of the NLRP3 inflammasome (Wu et al., 2024). These actions collectively suppress the pro-inflammatory M1 polarization of macrophages/microglia and inhibit a form of inflammatory cell death known as pyroptosis, contributing to improved functional recovery in models of cerebral and myocardial IRI (Ran et al., 2021; Wang et al., 2019).

#### 4.1.3 Inhibition of regulated cell death: apoptosis, ferroptosis, and autophagy

Accumulating evidence indicates that curcumin protects against IRI-induced cell loss by targeting multiple death pathways. It has been shown to inhibit the mitochondrial intrinsic apoptotic pathway by modulating the Bcl-2 family protein balance (increasing Bcl-2/Bax ratio) and suppressing the activation of executioner caspase-3, thereby reducing apoptotic cells in heart, kidney, and neuronal tissues (Yuan et al., 2024; Yang et al., 2021; Zhang Y. et al., 2018). In models of myocardial IRI, curcumin has been found to counteract ferroptosis—an iron-dependent form of cell death—by reducing intracellular iron and lipid peroxidation while upregulating the key anti-ferroptotic protein GPX4 and improving the GSH/GSSG ratio. This effect is potentially mediated through the upregulation of HES1 and activation of AMPK signaling (Yuan et al., 2024). Further studies in myocardial IRI indicate that curcumin suppresses autophagy-dependent ferroptosis and apoptosis via the Sirt1/AKT/FoxO3a pathway, leading to improved cardiac function (Zhao et al., 2025). Curcumin has also been reported to modulate excessive autophagy induced by IRI in cardiac and cerebral models, where injury typically upregulates LC3-II/LC3-I ratio and Beclin-1 while reducing p62, which correlates with reduced apoptosis and tissue damage (Yuan et al., 2024; Li X. et al., 2023; Zhang Y. et al., 2018; Huang et al., 2015). Additionally, curcumin has been demonstrated to attenuate endoplasmic reticulum (ER) stress triggered by IRI. It reduces the expression of ER stress markers like CHOP (C/EBP homologous protein) and caspase-11/12, helping to restore cellular homeostasis and promote survival (Zhang Y. et al., 2018; Chen et al., 2022; Sun et al., 2025).

#### 4.1.4 Protection of the microvascular endothelium and perfusion

In peripheral organs such as the liver, kidney and heart, curcumin has been reported to improve endothelial function and microvascular perfusion, in part by suppressing adhesion molecules (ICAM-1, VCAM-1, P- and E-selectin), thereby attenuating neutrophil–endothelial interactions and microvascular inflammation (Mohamadian et al., 2022; Bavarsad et al., 2019; Cui et al., 2021; Li et al., 2017). These vascular-protective effects has been shown to translate into better organ performance, including lower serum creatinine and BUN and improved renal hemodynamics in kidney IRI and sepsis-AKI models, as well as reduced cardiac enzyme release and infarct size with improved cardiac function in myocardial IRI models (Liu et al., 2017; Zeng et al., 2023; Wang et al., 2020; Machado et al., 2022; Wang et al., 2021; Bi et al., 2023).

### 4.2 Potential mechanisms of action in immunomodulation and chronic allograft injury

Allograft rejection is initiated when donor-derived alloantigens are recognized by the recipient immune system. This triggers both innate and adaptive immune responses. In the innate arm, dendritic cells (DCs) and macrophages capture and process donor antigens, promoting local inflammation and T-cell priming. Macrophages undergo pro-inflammatory M1 polarization, further amplifying the inflammatory response. These activated antigen-presenting cells then instruct the adaptive arm: recipient T cells recognize donor HLA antigens via direct, indirect, and semi-direct pathways, leading to T-cell activation, clonal expansion, and acute cellular rejection. Concurrently, B cells differentiate into plasma cells and produce donor-specific antibodies, which bind to graft endothelium, activate complement, and drive antibody-mediated rejection. Over time, persistent immune injury leads to chronic rejection, characterized by fibrosis, vasculopathy, and progressive graft dysfunction (Li and Lan, 2023). Curcumin has been reported to modulate several of these pathways (Figure 3), as discussed in the following sections.

**FIGURE 3:**
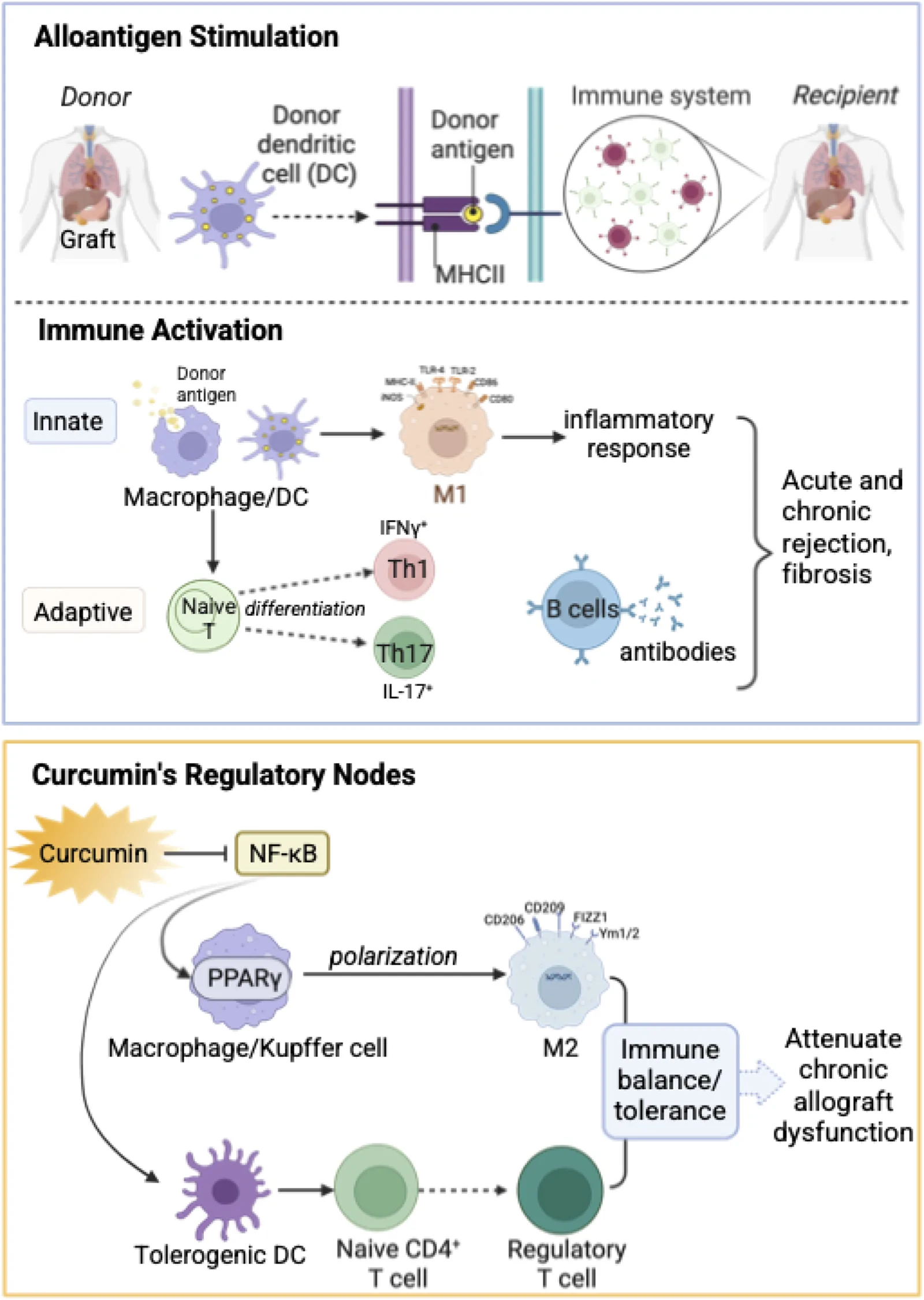
Curcumin’s potential immunomodulatory mechanisms in allograft rejection. Alloantigen stimulation triggers innate immune activation and adaptive immune responses, leading to acute rejection and chronic fibrotic injury (blue box). Curcumin has been reported to interfere with these processes through multiple mechanisms: suppressing NF-κB signaling pathway, and promoting macrophage M1-to-M2 repolarization, inducing tolerogenic dendritic cells, enhancing regulatory T-cell differentiation (orange box). These actions may shift the immune balance from a rejection-prone towards a regulatory state, potentially attenuating both acute rejection and chronic allograft dysfunction. Created with BioRender.com.

#### 4.2.1 Regulation of innate immunity: macrophage/kupffer cell polarization

The resident macrophages of the liver, kupffer cells (KCs), are pivotal initiators of IRI and inflammation post-transplantation (Zhai et al., 2011). In an orthotopic liver transplantation model, pre-treatment with curcumin significantly reduced intrahepatic production of ROS and pro-inflammatory cytokines while upregulating anti-inflammatory mediators. Mechanistically, curcumin was shown to activate PPARγ in KCs, which in turn inhibited NF-κB pathway (Liu et al., 2018). This shift promoted a phenotypic transition from the pro-inflammatory M1 state to the reparative M2 state, resulting in attenuated hepatocellular damage and improved post-transplant survival (Liu et al., 2018). This M1-to-M2 repolarization effect is corroborated by *in vitro* studies across various inflammation models, in which curcumin has been shown to suppresse the TLR4–MAPK–NF-κB axis, downregulating M1 markers (iNOS, TNF-α, IL-6, IL-12) while promoting expression of M2 markers (Arg-1, CD206) (Mohamm et al., 2019; Gao et al., 2015; Zhou et al., 2015; Abdollahi et al., 2023; Nasra et al., 2023).

#### 4.2.2 Shaping adaptive immunity: induction of tolerogenic dendritic cells and regulatory T cells

Curcumin critically influences the bridge between innate and adaptive immunity by modulating dendritic cell function. Studies have demonstrated that treatment with curcumin promotes a tolerogenic DC phenotype characterized by reduced surface expression of co-stimulatory molecules (CD80/CD86) and MHC class II, decreased production of IL-12, and impaired migratory capacity, while enhancing endocytic activity (Rahimi et al., 2020). These tolerogenic DCs have been found to exhibit a diminished ability to stimulate the proliferation of allogeneic T cells. More importantly, they skew T-cell differentiation by fostering the induction and expansion of CD4^+^CD25^+^Foxp3^+^ regulatory T cells (Tregs) while simultaneously inhibiting the polarization of pro-inflammatory T helper 1 (Th1) and T helper 17 (Th17) subsets (Rahimi et al., 2020; Ghoushi et al., 2024; Zhao et al., 2017). Supporting evidence comes from a murine model of acute graft-versus-host disease (GVHD), where curcumin treatment of donor splenocytes was found to suppress Th1/Th17 responses (reduced IFN-γ and IL-17), increase CD4^+^ and CD8^+^ Tregs, and consequent alleviate tissue damage in target organs (Park et al., 2013).

Despite these promising findings, significant knowledge gaps remain. Most studies have focused on T-cell-mediated responses, while the effects of curcumin on antibody-mediated rejection and donor-specific antibody production remain largely unexplored. Whether curcumin influences B-cell differentiation, plasma cell function, or complement activation in the transplant setting is currently unknown. Addressing these gaps will be essential to fully assess curcumin’s immunomodulatory potential in transplantation.

## 5 Potential interactions of curcumin with conventional immunosuppressants

The promising preclinical profile of curcumin naturally leads to the question of its potential integration into existing immunosuppressive regimens. A critical appraisal of its interactions—both synergistic and adverse—with drugs like mTOR inhibitors (mTORi), calcineurin inhibitors (CNIs), and corticosteroids is essential for any realistic clinical translation (Table 2). Current evidence, derived from limited *in vitro*, animal, and indirect clinical observations, paints a picture of both opportunity and significant caution.

**TABLE 2:** Summary of potential interactions between curcumin and common immunosuppressants in transplantation.

Immunosuppressant class	Primary mechanism of action	Nature of interaction with curcumin	Evidence level & key findings	Clinical implication & caution
mTOR Inhibitors	Inhibits mTOR pathway, blocking IL-2 driven T-cell proliferation	Pharmacodynamic SYNERGY (at optimal doses). Mechanism: Curcumin’s inhibition of NF-κB complements sirolimus’s mTOR inhibition for multi-pathway T-cell suppression	Limited *in vitro* evidence. • Synergistic inhibition of T-cell proliferation in PBMC models at low doses. • Effect may shift to additive/antagonistic at high doses	Potential for dose reduction? Theoretically promising for a “drug-sparing” strategy, but no *in vivo* or clinical data. Requires careful dose-finding
Calcineurin Inhibitors	Inhibits calcineurin, blocking IL-2 transcription	Pharmacokinetic RISK (Major). Mechanism: Curcumin may inhibit CYP3A4/P-gp, increasing CNIs blood levels. Pharmacodynamic: Theoretical organ-protection via anti-oxidant effects	Theoretical/Indirect clinical warning. • Turmeric/curcumin listed as potential CYP3A4/P-gp inhibitor. • No direct PK studies in transplant patients	High Risk of Toxicity. Mandatory Therapeutic Drug Monitoring if co-administered. Do not use without close supervision
Corticosteroids	Binds glucocorticoid receptor, suppressing broad inflammatory gene expression	Potential Local Steroid-Sparing and Receptor-level Additive Effect. Mechanism: Both activate GPR97 receptor via different sites; additive *in vitro*	Limited clinical evidence. • Topical curcumin comparable to topical steroids in oral GVHD. • No data in solid organ transplantation	Possible local alternative. May reduce topical steroid use. Systemic steroid-sparing effect unknown

### 5.1 Interaction with mTORi

In an *in vitro* model using human peripheral blood mononuclear cells, curcumin monotherapy inhibited OKT3-induced T-cell proliferation. When combined with low-dose sirolimus, a clear synergistic immunosuppressive effect was observed, achieving near-complete proliferation inhibition at lower concentrations of both agents (Deters et al., 2013). This represents the most compelling evidence for pharmacodynamic synergy exists with mTORi. However, this synergy appears concentration-dependent, as increasing the dose of either drug shifted the effect towards additivity or even potential antagonism, highlighting the existence of a narrow optimal therapeutic window (Deters et al., 2013). Mechanistically, sirolimus primarily inhibits the mTOR pathway, blunting IL-2-driven T-cell proliferation. Curcumin, by concurrently suppressing upstream pathways like NF-κB, may provide multi-targeted inhibition of T-cell activation and cytokine production, potentially creating a more robust immunosuppressive effect (Klawitter et al., 2015). Clinically, mTORi are used in combination with CNIs to allow CNIs dose reduction and mitigate nephrotoxicity (Grigg et al., 2019). This raises a theoretical possibility of a “triple-drug-sparing” strategy, where the addition of curcumin to an mTORi-based regimen could permit further reduction of mTORi or CNIs doses. However, no direct animal or clinical data currently exist to validate this hypothesis.

### 5.2 Interaction with CNIs

When curcumin is co-administered with calcineurin inhibitors, a potential pharmacokinetic interaction warrants consideration. Tacrolimus and cyclosporine are primarily metabolized by CYP3A4 and effluxed by P-glycoprotein (P-gp) (Vanhove et al., 2016). Curcumin and turmeric extracts have been classified as potential inhibitors of both CYP3A4 and P-gp (Vanhove et al., 2016)^,^ (Miedziaszczyk et al., 2022). While direct human interaction studies in transplant patients are scarce, this profile places curcumin on the list of dietary constituents that may elevate CNIs blood concentrations, increasing the risk of nephrotoxicity and other adverse effects. This would necessitate strict therapeutic drug monitoring if high-dose or high-bioavailability curcumin formulations are used concomitantly. CNIs toxicity (nephrotoxicity, hypertension, diabetes) is linked to oxidative stress and inflammation (Diker Cohen et al., 2025). Given curcumin’s established antioxidant and anti-inflammatory properties, there is a theoretical basis for it to mitigate some CNIs-induced organ damage (Wang X. et al., 2024). Nevertheless, no evidence from transplant populations suggests that curcumin can safely enable CNIs dose reduction or significantly attenuate its toxicity profile.

### 5.3 Interaction with corticosteroids

Both curcumin and glucocorticoids can activate G protein-coupled receptor (GPR97), albeit through different structural regions of the receptor, and their combined activation exhibits an additive effect *in vitro* (Harada et al., 2022). This suggests a potential for synergistic immunomodulation in certain pathways, though this has not been tested in transplant models. More concrete, though limited, evidence comes from the management of graft-versus-host disease (GVHD). In patients with oral GVHD following allogeneic hematopoietic stem cell transplantation, topical curcumin patches demonstrated efficacy comparable to topical triamcinolone (a corticosteroid) in improving lesions and pain (Mansourian et al., 2017). This indicates that curcumin may serve as a local steroid-sparing agent in mucosal inflammation, potentially reducing long-term exposure to topical steroids.

## 6 Challenges and future directions

Despite the compelling preclinical evidence, translating curcumin into a viable intervention for organ transplantation faces significant challenges. Strategically addressing these hurdles is paramount.

### 6.1 Bridging the gap between biological promise and translational reality

The most prominent obstacle to curcumin's clinical application is its inherently poor pharmacokinetic profile, characterized by extremely low aqueous solubility, inadequate gastrointestinal absorption, 386 rapid metabolism, and swift systemic clearance (Wang et al., 2025b; Dei Cas and Ghidoni, 2019). This limits its effective delivery from diet or standard supplements. Consequently, curcumin has sparked controversy regarding its potential as a 388 pharmaceutical agent (Baell and Michael, 2014; Heger et al., 2017; Nelson et al., 2017). Critics have labeled it a “pan-assay interference compound” (PAINS) (Baell and Michael, 2014) or an “invalid metabolic panacea” (IMPS) (Bisson et al., 2016), citing its low bioavailability, potential for 390 assay artifacts, and the disconnect between preclinical effects and negligible systemic exposure.

Rather than dismissing curcumin based on these valid critiques, the field has shifted to advanced formulation strategies. Major strategies include nanotechnology (liposomes, nanoparticles) (Tabanelli et al., 2021; Yakubu and Pandey, 2024; Karthikeyan et al., 2020), phospholipid complexation (phytosomes) (Zhang et al., 2025; Liu et al., 2020), structural analogs (Chelimela et al., 2024), and absorption enhancers like piperine. The validity of this formulation-based approach is supported by regulatory progress, such as the GRAS designation for some dietary formulations (Dei Cas and Ghidoni, 2019; Sharifi-Rad et al., 2020), and by clinical trial evidence showing that enhanced bioavailability correlates with efficacy signals (Hegde et al., 2023).

However, a critical gap remains for transplantation. Most advanced formulations lack the human pharmacokinetic, long-term safety data, and regulatory status needed for clinical use (Yakubu and Pandey, 2024; Karthikeyan et al., 2020; Stohs et al., 2020; Jacob et al., 2024). In the specific context of solid organ transplantation, the only human data come from a combination product without pharmacokinetic monitoring, perioperative physiology alters drug distribution unpredictably, and acute protection requires high tissue concentration precisely at the moment of reperfusion. Transplant-specific, clinical-grade formulations for perioperative or chronic use alongside immunosuppressants are still underdeveloped.

### 6.2 Designing a translational research pathway

Translating curcumin’s pleiotropic activity requires a deliberately staged research pathway integrating mechanism, pharmacology, and rigorous outcomes (Figure 4). Based on the current evidence, curcumin could potentially be integrated into transplant strategies at three levels: as an additive to preservation solutions to reduce initial graft injury; as a perioperative adjunct to modulate IRI; and as a chronic maintenance therapy to reduce immunosuppressant-related toxicities. Currently, only the first strategy has direct preclinical support, while the latter two require further formulation and safety studies.

**FIGURE 4:**
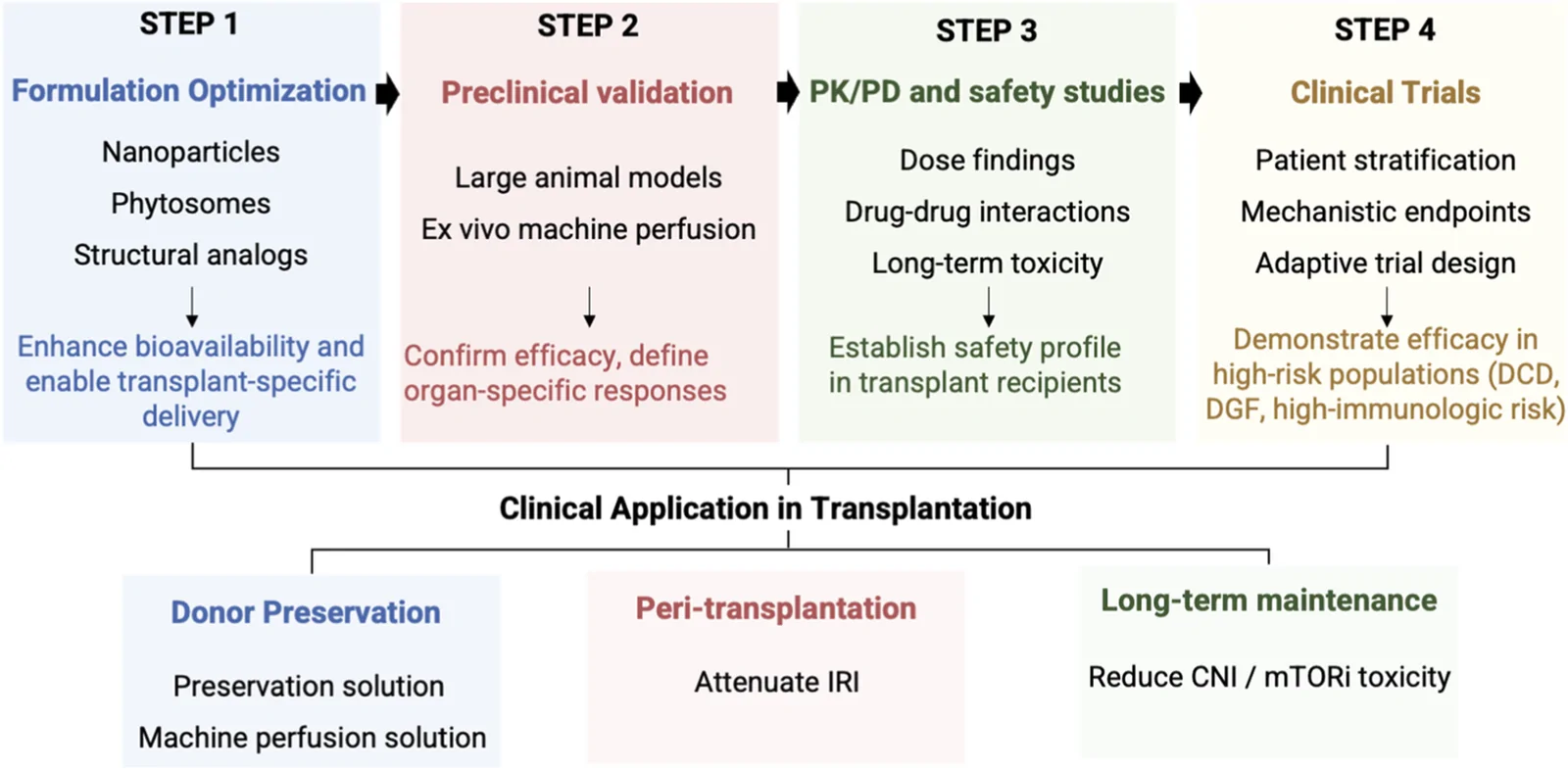
Proposed translational roadmap for curcumin in organ transplantation. Created with BioRender.com.

#### 6.2.1 Conducting mechanistically-informed clinical trials

Early-phase trials in transplantation should be explicitly hypothesis-driven, focusing on clearly defined mechanistic targets, such as suppression of NF-κB/JAK-STAT–mediated inflammation, attenuation of oxidative stress, or modulation of alloreactive T-cell responses, which are already well documented in preclinical and clinical contexts (Panknin et al., 2023). Patient enrollment should be enriched for populations in which IRI and inflammatory burden are highest, for example, recipients of marginal or DCD organs, those at high risk of delayed graft function, or high immunologic-risk recipients. Endpoints need to move beyond isolated surrogate markers to encompass clinically definitive outcomes, including the incidence and severity of PGD, biopsy-proven acute rejection rates, and trajectories of graft function, while embedding validated biomarkers of target engagement such as *ex vivo* T-cell assays, cytokine/oxidative stress signatures, and non-invasive injury markers (Hirt-Minkowski et al., 2023).

#### 6.2.2 Defining the protocol via integrated PK/PD studies

Despite extensive human experience with oral curcumin, fundamental questions regarding the optimal dose, formulation, timing, and route of administration for transplant recipients remain unresolved. Enhanced-bioavailability formulations can substantially increase systemic and tissue exposure, but their pharmacokinetics in immunosuppressed patients have not been systematically characterized. Dedicated PK/PD studies are therefore needed to define exposure-response relationships for bio-enhanced curcumin in the transplant setting, including peri-transplant and maintenance dosing schedules, and to systematically assess PK/PD drug-drug interactions with cornerstone immunosuppressants such as calcineurin and mTOR inhibitors. Such studies should explicitly distinguish unconjugated from curcumin species, given that biological activity *in vitro* typically requires micromolar concentrations of the parent compound, whereas most oral formulations yield only low-nanomolar plasma levels (Kroon et al., 2025).

#### 6.2.3 Establishing long-term safety and efficacy

Multiple dose-escalation and disease-specific trials have established a favorable short-to medium-term safety profile for curcumin. However, the long-term safety of chronic administration of high-exposure nano- or phospholipid-based formulations in patients on lifelong, multi-drug immunosuppression is unknown. Future studies must therefore extend follow-up beyond early post-transplant outcomes to evaluate effects on chronic allograft injury, including interstitial fibrosis/tubular atrophy and vasculopathy, as well as infection risk, malignancy, and metabolic complications (Karimi et al., 2022). Embedding histologic (protocol biopsies) and non-invasive fibrosis markers will be critical to determine whether curcumin can modify long-term allograft remodeling rather than merely blunt early IRI.

#### 6.2.4 Embracing divergent data and context-dependent biology

Curcumin research across liver injury, acute kidney injury, cancer, and critical illness consistently reveals context- and dose-dependent biology, with anti-apoptotic, cytoprotective effects in injured parenchyma but pro-apoptotic actions in proliferative or malignant cells (Liu et al., 2025; Wang Y. et al., 2024). Likewise, transplant-relevant data already include both positive findings—such as improved early renal allograft function in a curcumin-quercetin trial—and neutral results, as seen with cyclodextrin-curcumin added to preservation solution in a porcine DCD liver model (Shoskes et al., 2005; Meurisse et al., 2024). Rather than discarding such “negative” or divergent data, future work should systematically map how dose, formulation, timing (pre- or post-ischemia), disease stage, and tissue context shape curcumin’s effects on cell death, inflammation, and immune activation. Defining this multidimensional therapeutic window, and explicitly reporting neutral or harmful outcomes, will be essential to avoid overgeneralization from single disease models and to design rational, safe protocols for transplant recipients.

### 6.3 Insights from broader transplantation contexts

To fully explore curcumin’s potential in solid organ transplantation, insights from its application in other graft contexts can be instructive. Researches in tissue transplantation, such as islet (Pathak et al., 2016; Yang et al., 2025) and ovarian (Hendarto et al., 2025; Baki et al., 2024), provide models to study its direct cytoprotective effects on specific parenchymal cells, free from the vascular complexity of whole organs. Similarly, its efficacy in modulating GVHD following hematopoietic cell transplantation offers a clear paradigm of systemic immunomodulation in clinically relevant human setting (Palaniyandi et al., 2017). These parallel lines of investigation not only validate curcumin’s pleiotropic mechanisms in different biological systems but also present alternative methodological frameworks and combinatorial strategies (such as with cellular therapies (Guo et al., 2020; Li Y. et al., 2023)) that could be creatively translated to overcome the specific hurdles in solid organ transplantation.

## 7 Conclusion

Curcumin occupies an intriguing yet challenging position in transplantation research. Its pleiotropic pharmacology—antioxidant, anti-inflammatory, and immunomodulatory—aligns with the multifactorial nature of graft IRI and chronic dysfunction. However, its pharmacokinetic limitations represent a major translational hurdle that has yet to be overcome.

The evidence base remains fragmented. Kidney transplantation has yielded early clinical signals, liver research offers mechanistic depth but also a negative large-animal study, while heart and lung evidence is sparse. These gaps do not negate curcumin’s potential but expose the inadequacy of current research paradigms.

Moving forward, the field must pivot from testing native curcumin to developing transplant-optimized formulations, systematically evaluating drug-drug interactions, and conducting mechanism-driven trials targeting high-risk scenarios. If these hurdles are overcome, curcumin could emerge as a valuable adjunct in low-toxicity immunosuppressive regimens. Its story offers a broader lesson: for pleiotropic natural products, clinical success depends less on biological promise and more on the rigor with which formulation and pharmacokinetic challenges are addressed.
